# Diagnostic and prognostic comparison of stress electrocardiogram, cardiovascular magnetic resonance, and single photon emission computed tomography, alone and sequentially, in stable chest pain

**DOI:** 10.1016/j.jocmr.2025.101960

**Published:** 2025-09-15

**Authors:** Giandomenico Bisaccia, Peter P. Swoboda, John F. Younger, Neil Maredia, Catherine J. Dickinson, Julia M. Brown, Chiara Bucciarelli-Ducci, Sven Plein, John P. Greenwood

**Affiliations:** a Royal Brompton and Harefield Hospitals, Guys’ & St Thomas’ NHS Trust, London, UK; b Department of Neuroscience, Imaging and Clinical Sciences, “G. d′Annunzio” University of Chieti-Pescara, Chieti, Italy; c Leeds Institute of Cardiovascular and Metabolic Medicine, University of Leeds, Leeds, UK; d Royal Brisbane and Women’s Hospital, University of Queensland, Herston, QLD, Australia; e James Cook University Hospital, Middlesbrough, UK; f Department of Cardiology, Leeds Teaching Hospitals NHS Trust, Leeds, UK; g Leeds Institute of Clinical Trials Research, University of Leeds, Leeds, UK; h School of Biomedical Engineering and Imaging Sciences, Faculty of Life Sciences and Medicine, King’s College University, London, UK; i Baker Heart and Diabetes Institute and Monash University, Melbourne, Australia

**Keywords:** Angina, stable, Magnetic resonance imaging, Exercise test, Radionuclide imaging, Diagnostic techniques, cardiovascular, Prognosis

## Abstract

**Background:**

Exercise electrocardiogram (ECG) remains widely performed in the assessment of patients with suspected cardiac chest pain. We aimed to assess the comparative diagnostic and prognostic yield of exercise ECG, single photon emission computed tomography (SPECT), and cardiovascular magnetic resonance (CMR), in a large prospective patient population.

**Methods:**

Patients recruited to Clinical Evaluation of MAgnetic Resonance in Coronary heart disease (CE-MARC) who had exercise ECG were included and followed up to a median (interquartile range) of 6.3 (0.1, 6.8) years. Sensitivity, specificity, positive predictive value (PPV), negative predictive value (NPV), and area under the curve (AUC) for diagnostic accuracy were derived and hazard ratios of major adverse cardiovascular events (MACE) for prognostic significance were calculated.

**Results:**

Of 752 patients in the CE-MARC trial, 580 had exercise ECG and invasive coronary angiography, of which 503 also had SPECT and CMR. At follow-up, a total of 91 (15.7%) patients experienced MACE. Using invasive angiography as the reference test, the sensitivity, specificity, PPV, and NPV (95% confidence interval) of exercise ECG were 68.3 (61.9, 74.0), 72.5 (67.6, 76.9), 61.0 (54.8, 66.8), 78.4 (73.7, 82.5). Exercise ECG was significantly less sensitive than CMR and less specific than both CMR and SPECT. A positive exercise ECG result was not predictive of MACE at follow-up (Hazard ratio 1.14 [0.75, 1.72], p = 0.53). CMR had both a greater diagnostic and prognostic yield than exercise ECG, SPECT, and their combination. Sequential CMR following inconclusive exercise ECG was comparable to CMR alone as the first-line test.

**Conclusion:**

In patients with suspected angina, CMR alone as the first-line test was more sensitive and prognostically accurate than exercise ECG, SPECT, or sequential combination of both tests.

## Graphical abstract

**Figure:**
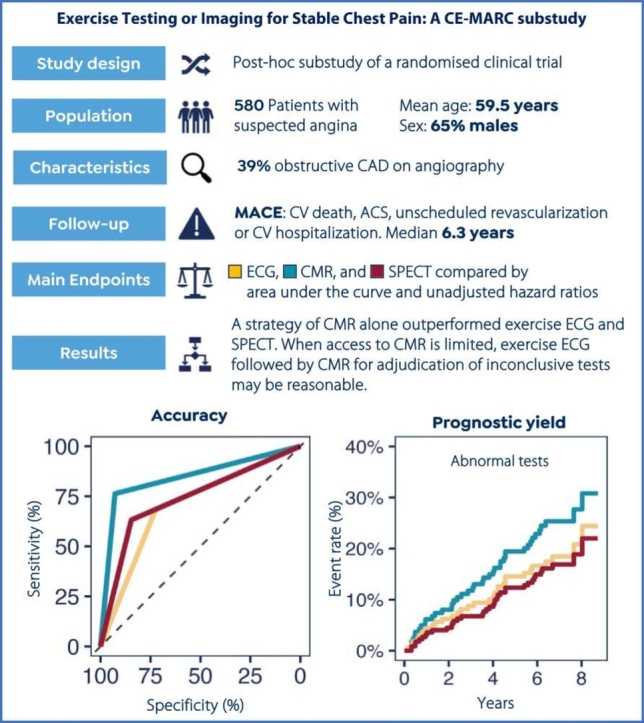
At head-to-head comparison, exercise ECG offered limited accuracy and no prognostic ability. A strategy of exercise ECG followed by CMR for inconclusive tests had comparable accuracy to a strategy of CMR alone. CMR outperformed exercise ECG, SPECT, and a combination of both.

## 1 Introduction

Several investigations are available for the diagnosis and risk stratification of patients with suspected cardiac chest pain 1, 2, 3, 4, 5. The 2021 AHA/ACC/ASE/CHEST/SAEM/SCCT/SCMR Guideline for the Evaluation and Diagnosis of Chest Pain recommends exercise electrocardiogram (ECG) testing and non-invasive imaging as potential first-line tests, but acknowledges that exercise ECG has a lower sensitivity than cardiac imaging to detect coronary artery disease (CAD) [6]. Despite known limitations around diagnostic accuracy, exercise ECG continues to be commonly utilized in the United States [7] due to widespread availability, lower cost, and its ability to assess a patient’s functional capacity, which is associated with prognosis. However, the diagnostic value of the exercise ECG can be limited by frequent inconclusive results, for example, when the predicted heart rate is not achieved, when exercise is limited by co-morbidities, when there are resting ECG abnormalities, or ECG changes are equivocal 8, 9. Current US guidelines recommend that, following an equivocal exercise ECG, a subsequent cardiac imaging test should be considered [6].

The sequential use of diagnostic tests for the detection of obstructive CAD has not been extensively evaluated in large-scale clinical trials. In particular, there is a paucity of data directly comparing exercise ECG with functional imaging tests in the same population [10]. Furthermore, minimal data exist on the diagnostic performance of exercise ECG compared with stress cardiovascular magnetic resonance (CMR) imaging.

The CE-MARC (Clinical Evaluation of MAgnetic Resonance in Coronary heart disease) trial was designed to establish the diagnostic performance of CMR in patients with suspected stable angina in comparison with myocardial perfusion scintigraphy by single photon emission computed tomography (SPECT) [11] in comparison to invasive coronary angiography; an exercise ECG was performed by protocol if patients were considered able to exercise. We report on the diagnostic and prognostic performance of exercise ECG compared with SPECT and CMR from the original CE-MARC trial population and examine the clinical utility of the sequential use of exercise ECG followed by either functional imaging test.

## 2 Methods

### 2.1 Design

The design and primary outcome of CE-MARC have been published 11, 12, 13. CE-MARC studied patients referred to cardiologists with suspected angina pectoris. The study was performed in accordance with the Declaration of Helsinki (2000), approved by the local Research Ethics Committee, and all patients provided written informed consent. Per protocol, an exercise ECG was mandated in all patients considered safely able to perform the test. All patients then underwent SPECT and CMR in a random order, followed by X-ray coronary angiography, regardless of the treating cardiologist’s clinical management intension or prior test results. Stratified permuted block randomization was used to ensure the groups were balanced for age (<65, ≥65 y) and sex. The SPECT test result was provided upon request to the referring clinician if SPECT would have been performed had the patient not participated in the trial; to avoid potential bias toward the investigation test (CMR), the CMR result was not disclosed. Patients were followed up for major adverse cardiovascular events (MACE, cardiovascular [CV] death, acute coronary syndrome, unscheduled revascularization, or hospital admission for CV cause). A CONSORT reporting checklist [14] is available in the Supplementary Material.

### 2.2 Patients

Patients were recruited between March 2006 and August 2009 from two UK secondary and tertiary care hospitals. Consecutive patients were screened and enrolled if they had at least one major CV risk factor and a cardiologist considered them to have possible stable angina requiring further cardiac investigation in accordance with contemporary clinical practice. Patients were classified into very low, low, intermediate, and high risk profiles, as per ACC/AHA guidelines [1]. Exclusion criteria were previous coronary artery bypass surgery, crescendo angina or acute coronary syndrome, contraindication to CMR (e.g., pacemaker) or adenosine infusion (reversible airways disease, atrioventricular heart block), pregnancy, inability to lie supine, and estimated glomerular filtration rate ≤30 mL/min/1.73 m^2^.

### 2.3 Investigational procedures and their analysis

Detailed procedural protocols have been published [11]. A brief description is given below.

*Exercise ECG*: Exercise ECG was performed with treadmill testing using a Bruce or modified Bruce protocol. All exercise ECGs were supervised and reviewed by experienced technical staff. The test report contained the protocol used, resting heart rate and blood pressure (BP), maximum achieved heart rate and BP, if 85% maximum predicted heart rate for age and sex achieved, duration, symptoms, whether limiting angina occurred and the reason for stopping (chest pain, breathlessness, arrhythmia, leg pain, ST change, fatigue, target heart rate achieved, hypo or hypertension), maximum ST segment deviation, time to this, and the heart rate after 1 min of recovery. Findings of the exercise ECG were reviewed by the cardiologist responsible for the patient and used to classify the test as positive, negative, or inconclusive. A positive test was where there was ≥1 mm of horizontal or downsloping ST segment depression or elevation for at least 60 to 80 ms after the end of the QRS complex as recommended in the AHA/ACC guidelines [1]. A negative test was the absence of ST change with adequate exercise duration and heart rate response. An inconclusive test was where the ST segment change failed to reach at least 1 mm or the exercise time or heart rate response was deemed inadequate. The Duke Treadmill Score was also calculated for each study case.

*SPECT*: SPECT radionuclide imaging used a dedicated cardiac gamma camera (MEDISO Cardio-C, Budapest, Hungary). Patients underwent a 2-day protocol using 99mTc tetrofosmin (Myoview, GE Healthcare, Little Chalfont, Buckinghamshire, UK), standard dose of 400 MBq adjusted by weight to a maximum 600 MBq per examination (effective dose 6–9 mSv). Stress and rest ECG-gated SPECT images were acquired with an intravenous adenosine protocol (140 μg/kg/min for 4 min). SPECT data were analyzed visually, by blinded paired readers with at least 10 y experience using the standard 17-segment AHA/ACC model. SPECT results were deemed positive with evidence of ischemia by visual or semi-quantitative myocardial perfusion methods, and/or with evidence of regional wall motion abnormalities. After X-ray angiography, the SPECT result could, on request, be made available to facilitate a revascularization decision (it was considered unethical by the ethics committee to blind the treating clinician to this result if requested).

*CMR*: The multi-parametric CMR (1.5T Philips Intera CV scanner; Philips Healthcare, Best, The Netherlands) protocol comprised adenosine stress perfusion, magnetic resonance (MR) coronary angiography, rest perfusion, cine imaging for left ventricular function, and late gadolinium enhancement (LGE). The pharmacological stress protocol was identical to SPECT. Analogous to SPECT, CMR data were analyzed blinded, by paired readers with at least 10 years’ experience using visual analysis according to the standard AHA/ACC 17-segment model. CMR was considered positive if any of the study components (myocardial perfusion, LGE, MR coronary angiography, and/or regional wall motion abnormalities) were deemed abnormal. CMR results were kept blind from clinicians as it was considered a research investigation.

*X-ray angiography*: All X-ray angiograms were performed after CMR and SPECT and reported by two experienced invasive cardiologists blinded to the other studies. Obstructive CAD was defined as ≥70% stenosis of a first-order coronary artery measuring ≥2 mm in diameter or left main stem stenosis ≥50%, by quantitative coronary angiography (QCA) using QCAPlus software (Sanders Data Systems, Palo Alto, California, USA), with a post-stenosis diameter used as the reference vessel diameter in cases of ostial disease.

### 2.4 Statistics

Baseline characteristics were summarized using descriptive statistics. The sensitivity, specificity, positive and negative predictive values (PPV, NPV) were determined, and corresponding 95% confidence intervals (CI) for the diagnostic performance of exercise ECG, CMR, and SPECT compared with the X-ray angiogram were calculated using the Wilson Score method. Patients were included in the analysis if they had complete data from both exercise ECG and X-ray angiography. Diagnostic performance of exercise ECG combined with SPECT or with CMR was compared in patients with evaluable results to both tests and to X-ray angiography. Performance of tests in the same population had sensitivity and specificity compared using McNemar’s test and predictive values using the Generalized Score Statistic. The performance of a single test in two separate subgroups (e.g., males and females) was compared using Pearson’s chi-squared test for sensitivity and specificity; analogous comparisons were not performed for NPV and PPV due to the difference in prevalence between the two sexes. Overall diagnostic accuracy of exercise ECG, CMR, and SPECT was compared by receiver operating characteristics (ROC) curves with results of each test considered as either positive, negative, or inconclusive. Additional ROC analyses with scored results (i.e., CMR and SPECT results labeled as normal, probably normal, compatible with ischemic heart disease, compatible with mild inducible ischemia, compatible with moderate/severe inducible ischemia, or inconclusive; exercise ECG results as positive, borderline positive, negative, or inconclusive) are presented in the Supplementary Material. Decision curve analysis was performed to evaluate the net clinical benefit of exercise ECG, CMR, and SPECT in reducing unnecessary invasive coronary angiography, after accounting for patients’ baseline risk as estimated by the ESC-pre-test probability (PTP) model [15]. Additional diagnostic evaluation of the exercise ECG by sex and pre-test clinical likelihood of CAD, along with an assessment of the diagnostic and prognostic value of the Duke Treadmill Score, is available in the Supplementary Material.

Kaplan-Meier curves were used to estimate survival probabilities and compare MACE rates among patients with different test results. The log-rank test was used to compare survival curves. Cox proportional hazards regression models were used to estimate unadjusted hazard ratios (HRs) and 95% CIs for the association between test results and MACE. The proportional hazards assumption was tested using Schoenfeld residuals. Statistical analysis was undertaken using R version 4.1.0 (R Foundation for Statistical Computing, Vienna, Austria) at a two-sided 5% significance level.

## 3 Results

**Fig. 1:**
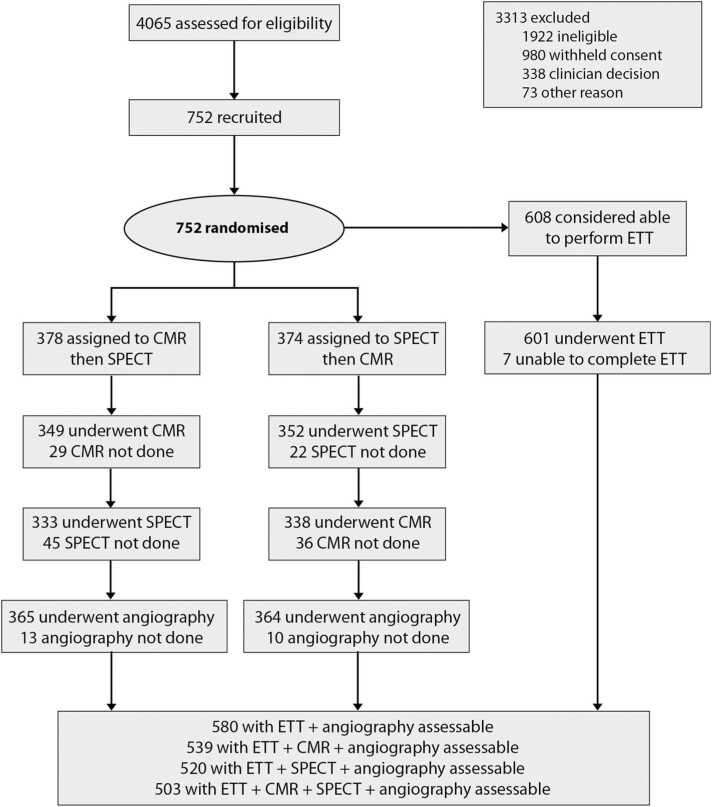
Patient flow diagram. *CMR* cardiovascular magnetic resonance, *SPECT* single photon emission computed tomography, *ETT* exercise treadmill test.

Of 752 patients randomized in the CE-MARC trial, 608 were considered by the treating cardiologist to be able to exercise at the point of recruitment/consent. Among these, 7 patients were unable to complete the exercise ECG protocol (Fig. 1), leaving 601 patients for this analysis. The modified Bruce protocol was used in 13 (2.2%) patients.

Five hundred and eighty of the 601 patients with exercise ECG results available had invasive coronary angiography. Table 1 shows the demography of these 580 patients compared with (1) the 752 randomized, and those groups having (2) exercise ECG and SPECT (n = 520), and (3) exercise ECG and CMR (n = 539). A total of 503 patients underwent all four study tests (exercise ECG, SPECT, CMR, and coronary angiography).

**Table 1:** Demography of the whole study population and those undergoing non-invasive testing in addition to X-ray angiography.

		All randomized patients	Patients with exercise ECG and X-ray angio	Patients with X-ray angio, exercise ECG, and SPECT results	Patients with X-ray angio, exercise ECG, and CMR results
Variable		(N=752)	(N=580)	(N=520)	(N=539)
Age (y)	Mean (SD)	60.2 (9.7)	59.5 (9.6)	59.5 (9.5)	59.6 (9.6)
Sex	Male	471 (63%)	375 (65%)	338 (65%)	347 (64%)
Body mass index	Mean (SD)	29.2 (4.4)	28.9 (4.3)	28.8 (4.2)	28.8 (4.2)
Ethnicity	White	711 (95%)	549 (95%)	494 (95%)	512 (95%)
	Black	6 (1%)	6 (1%)	5 (1%)	5 (1%)
	Asian	30 (4%)	22 (4%)	18 (4%)	19 (4%)
	Other	5 (1%)	3 (1%)	3 (1%)	3 (1%)
Smoking status	Never smoked	257 (34%)	208 (36%)	188 (36%)	196 (36%)
	Ex-smoker	350 (47%)	272 (47%)	244 (47%)	253 (47%)
	Current smoker	145 (19%)	100 (17%)	88 (17%)	90 (17%)
Systolic BP (mmHg)	Mean (SD)	137.9 (20.7)	137.6 (20.1)	137.2 (20.6)	137.6 (20.4)
Diastolic BP (mmHg)	Mean (SD)	79.0 (11.3)	79.2 (10.5)	79.0 (10.6)	79.2 (10.6)
Previous AMI or ACS		60 (8%)	33 (6%)	30 (6%)	32 (6%)
Previous PCI		38 (5%)	16 (3%)	14 (3%)	16 (3%)
Hypertension		394 (52%)	293 (51%)	253 (49%)	266 (49%)
Diabetic		96 (13%)	59 (10%)	57 (11%)	57 (11%)
	Type I	4 (4%)	4 (7%)	4 (7%)	4 (7%)
	Type II	92 (96%)	55 (93%)	53 (93%)	53 (93%)
Family history of premature heart disease	Yes	430 (57%)	332 (57%)	300 (58%)	311 (58%)
	No	268 (36%)	204 (35%)	181 (35%)	187 (35%)
	Unknown	54 (7%)	44 (8%)	39 (8%)	41 (8%)
Total cholesterol (mmol/L)	Mean (SD)	5.2 (1.2)	5.3 (1.2)	5.3 (1.2)	5.3 (1.2)
Aspirin and/or clopidogrel		454 (60%)	340 (59%)	299 (58%)	313 (58%)
Statin		336 (45%)	226 (39%)	203 (39%)	210 (39%)
ACE inhibitors/angiotensin II		258 (34%)	175 (30%)	155 (30%)	164 (30%)
Blockers					
Nicorandil		14 (2%)	5 (1%)	4 (1%)	5 (1%)
Nitrates		322 (43%)	243 (42%)	213 (41%)	223 (41%)
Beta-blocker		235 (31%)	170 (29%)	143 (28%)	152 (28%)
Calcium antagonist		144 (19%)	83 (14%)	70 (13%)	73 (14%)
Risk of coronary artery disease					
	Low (PTP < 15%)	154 (20%)	128 (22%)	119 (22%)	117 (22%)
	Intermediate-high (PTP > 15%)	598 (80%)	452 (78%)	420 (78%)	422 (78%)
Any significant coronary artery stenosis		282 (39%)	224 (39%)	201 (39%)	211 (39%)

*ECG* electrocardiogram*, SPECT* single photon emission computed tomography*, CMR* cardiovascular magnetic resonance*, BP* blood pressure*, AMI* acute myocardial infarction*, ACS* acute coronary syndrome*, PCI* percutaneous coronary intervention*, PTP* pre-test probability*, SD* standard deviation

The prevalence of obstructive CAD at invasive angiography in the exercise ECG population was 39%, comparable to that in the original trial (Table 1) [12]. Additionally, based on an externally validated, contemporary PTP score, 405 (77.3%) patients had a CAD risk ≥15% and were therefore considered at intermediate-high risk, while 118 (22.7%) were considered at low risk. CV risk and extent of obstructive CAD in male and female patients having exercise ECG and coronary angiography are presented in Table S1.

### 3.1 Exercise ECG findings

Of those who performed the exercise ECG (n = 601), 64.7% of patients achieved at least 85% of the maximum predicted heart rate and the mean (SD) exercise time was 6.5 (2.9) min with a median of 6.3 (interquartile range [IQR] 4.4–8.7) min. Limiting angina was noted in 20.6%, non-limiting in 37.9%; breathlessness (25.8%) was the commonest reason to stop with fatigue given as the reason in 24.0%. The mean (SD) exercise time to maximum ST depression was 6.5 (3.0) min and the median 6.4 (IQR 4.5–8.3) min. Additional results from exercise ECG are presented in Table S2.

### 3.2 Diagnostic accuracy

#### 3.2.1 Exercise ECG

**Fig. 2:**
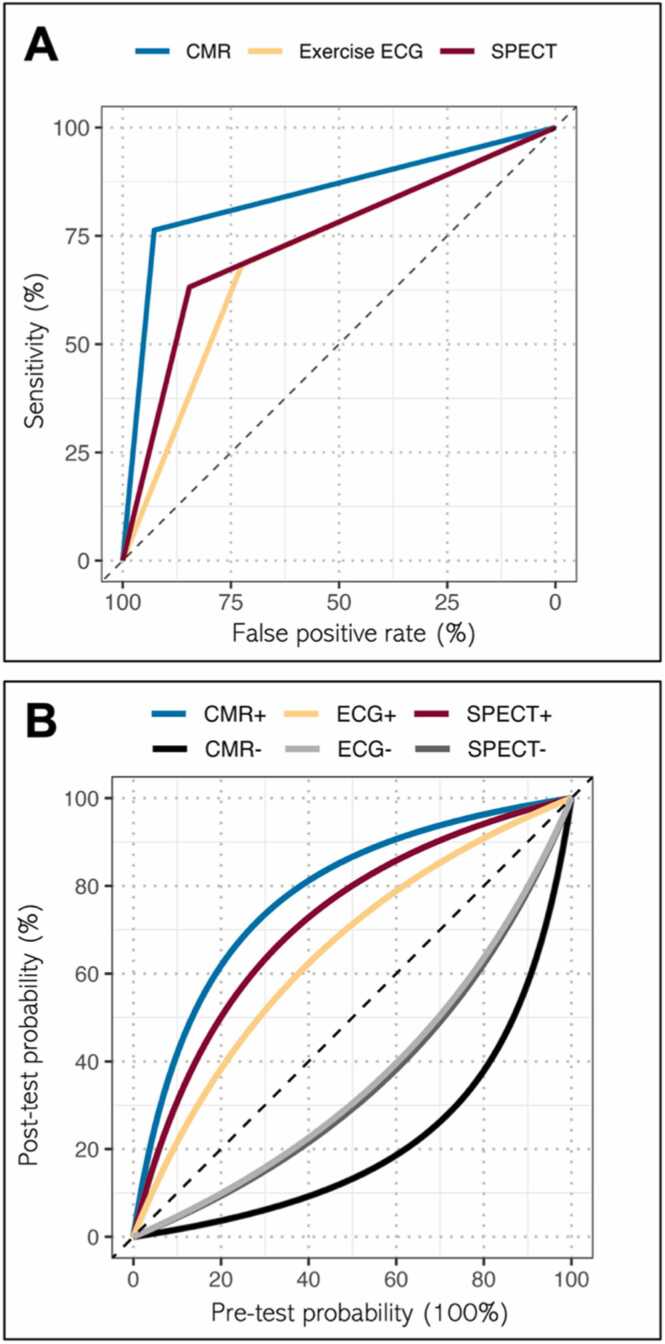
Diagnostic yield of exercise ECG, CMR, and SPECT in CE-MARC. (A) ROC curves demonstrating diagnostic accuracy of exercise ECG, CMR, and SPECT; (B) leaf plot based on CAD-PTP and result of each test. *ECG* electrocardiogram, *SPECT* single photon emission computed tomography, *CMR* cardiovascular magnetic resonance, *CE-MARC* Clinical Evaluation of MAgnetic Resonance in Coronary heart disease, *ROC* receiver operating characteristics, *CAD* coronary artery disease, *PTP* pre-test probability

In the 580 patients with invasive coronary angiography, the exercise ECG was positive in 251 (43.3%), negative in 73 (12.6%), and inconclusive in 256 (44.1%) patients. Compared with invasive angiography, the sensitivity, specificity, PPV, and NPV for exercise ECG when considering inconclusive tests as negative were 68.3%, 72.5%, 78.4%, and 61%, respectively (area under the curve [AUC] 70.4, Fig. 2). Exclusion of the 256 inconclusive tests resulted in a substantial increase in sensitivity but with loss of specificity, an unchanged PPV, and increase in NPV (AUC 68.9; p = 0.5986; Table S3 and Figure S1). These were consistent between male and female patients, and for patients at higher versus lower pre-test likelihood of CAD (Supplementary Results).

#### 3.2.2 CMR and SPECT

Accuracy metrics for the main trial population are previously published [12]. Of the 580 patients having exercise ECG and invasive angiography, 539 also had CMR and 520 had SPECT. In the 539 patients with CMR, the sensitivity, specificity, PPV, and NPV were all significantly lower (p < 0.0001) for exercise ECG compared to CMR (Table 2B). In the 520 patients with SPECT, the sensitivity and NPV were similar between the two tests but the specificity (p = 0.0003) and PPV (p = 0.0021) for exercise ECG were lower (Table 2D). By ROC curve comparison, CMR was found to allow better obstructive CAD discrimination than exercise ECG (p < 0.0001) and SPECT (p < 0.0001) (Fig. 2 and Tables S4 and S5). No significant difference was found in overall test accuracy between exercise ECG and SPECT (p = 0.2412). Analysis of ROC curves with scored results is presented in Figure S2.

**Table 2:** Diagnostic accuracy of exercise ECG against CMR and SPECT.

Test	Number of patients	Sensitivity, % (95%CI)	Specificity, % (95%CI)	Positive predictive value, % (95%CI)	Negative predictive value, % (95%CI)
A. Exercise ECG (inconclusive tests considered negative)	580	68.3	72.5	61.0	78.4
		(61.9, 74.0)	(67.6, 76.9)	(54.8, 66.8)	(73.7, 82.5)
A. Exercise ECG (patients with at least another test)	539	69.7	73.2	62.6	78.9
		(63.2, 75.5)	(68.1, 77.7)	(56.2, 68.5)	(74.0, 83.2)
B. CMR (in exercise ECG group)	539	87.2	86.9	81.1	91.3
		(82.0, 91.1)	(82.8, 90.1)	(75.5, 85.6)	(87.7, 94.0)
	A vs B	p < 0.0001	p < 0.0001	p < 0.0001	p < 0.0001
C. Exercise ECG	520	69.7	72.1	61.1	79.0
		(63.0, 75.6)	(66.9, 76.7)	(54.7, 67.2)	(74.0, 83.3)
D. SPECT (in exercise ECG group)	520	65.7	84.0	72.1	79.5
		(58.9, 71.9)	(79.6, 87.6)	(65.2, 78.1)	(74.9, 83.5)
	C vs D	p = 0.4222	p = 0.0003	p = 0.0021	p = 0.8331

*ECG* electrocardiogram*, SPECT* single photon emission computed tomography*, CMR* cardiovascular magnetic resonance*, CI* confidence interval

#### 3.2.3 Sequential use of SPECT or CMR after exercise ECG

We examined the sequential use of either SPECT or CMR in patients with inconclusive exercise ECG tests, accepting the exercise ECG results for the clearly positive and clearly negative tests (as per clinical practice). All four study tests (exercise ECG, CMR, SPECT, and coronary angiography) were undertaken in 503 patients. A strategy of sequential CMR after inconclusive exercise ECG had significantly superior sensitivity and NPV compared to sequential SPECT after exercise ECG (p = 0.0266 and p = 0.0114, respectively). A strategy of sequential SPECT after inconclusive exercise ECG improved the sensitivity compared to SPECT alone (65.5% to 86.3%) but with reduced specificity (83.7% to 64.1%). Correspondingly, the NPV improved, but with a fall in the PPV. For sequential use of CMR after inconclusive exercise ECG, compared to CMR alone, there was a small increase in sensitivity (86.8% to 91.9%) which corresponded to a reduction in specificity (86.6% to 66.0%); the NPV was comparable but there was a significant reduction in the PPV. Comprehensive results are presented in Table S3.

#### 3.2.4 Projected reduction in unnecessary coronary angiographies

Across the entire threshold probability range (15%–80%), CMR demonstrated the greatest incremental value over the ESC-PTP model alone, with an estimated 20–50 fewer unnecessary angiographies per 100 patients. SPECT also conferred a meaningful reduction, particularly at higher threshold probabilities, though its performance remained consistently below that of CMR. Exercise ECG provided only modest incremental benefit, with smaller reductions across the threshold spectrum (Fig. S3).

#### 3.2.5 Prognostic significance

At a median follow-up of 6.3 (IQR 0.1–6.8) y, 91 (15.7%) out of 580 patients experienced MACE. Survival rates based on results of CMR and SPECT in the whole trial population have been previously published [16], with CMR, but not SPECT, conferring additional risk stratification after adjustment for other CV risk factors.

#### 3.2.6 Exercise ECG

**Fig. 3:**
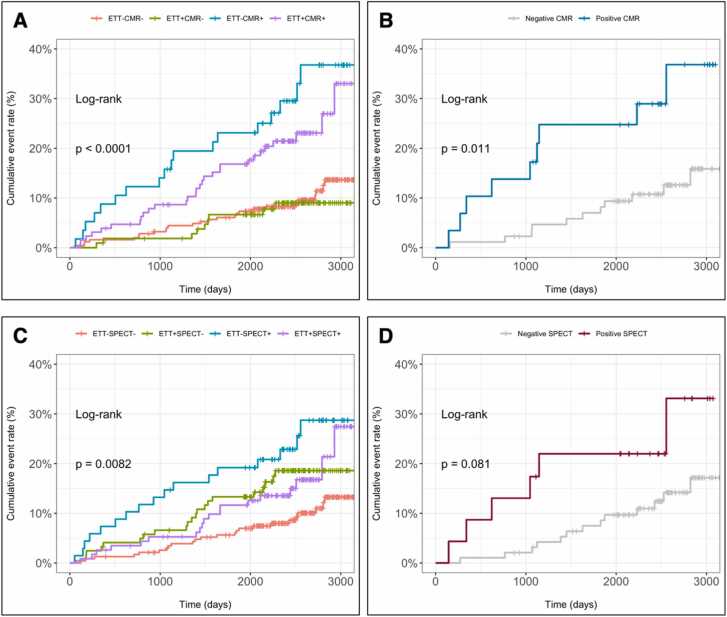
Prognostic significance of exercise ECG+CMR (A), CMR in patients with inconclusive exercise ECG (B), exercise ECG+SPECT (C), and SPECT in patients with inconclusive ECG (D). *ECG* electrocardiogram, *SPECT* single photon emission computed tomography, *CMR* cardiovascular magnetic resonance

Abnormal exercise ECG findings were not associated with increased risk of MACE (unadjusted HR, 1.14 [95%CI, 0.75–1.72]; p = 0.53) (Fig. 3A). This finding was also confirmed when inconclusive exercise ECG tests were excluded (HR, 2.09 [95%CI, 0.93–4.68]; p = 0.073) (Fig. 3B).

#### 3.2.7 Sequential use of imaging after exercise ECG

*Exercise ECG followed by CMR:* A positive CMR study was associated with increased risk of MACE both in patients with a negative (HR, 3.54 [95%CI, 1.94–6.47]; p < 0.001) and positive (HR, 2.52 [95%CI, 1.48–4.28]; p < 0.001) exercise ECG. Patients with a positive exercise ECG who had a negative CMR were not found to be at increased risk of MACE (HR, 0.84 [95%CI, 0.39–1.78]; p = 0.6418) (Fig. 3A). Among patients with an inconclusive exercise ECG, a positive CMR study significantly predicted MACE at follow-up (HR, 2.98 [95%CI, 1.23–7.20]; p = 0.015) (Fig. 3B).

*Exercise ECG followed by SPECT:* Abnormal SPECT findings had incremental prognostic ability in patients with a negative (HR, 2.79 [95%CI, 1.50–5.19]; p = 0.001), but not positive (HR, 1.78 [95%CI, 0.97–3.25]; p = 0.0617) exercise ECG (Fig. 3C). Compared with patients with both negative exercise ECG and SPECT, patients with a positive exercise ECG who had a negative SPECT were instead found to be at increased risk of MACE (HR, 1.89 [95%CI, 1.05–3.39]; p = 0.0340). A positive SPECT study was not significantly associated with MACE at follow-up among patients with an inconclusive exercise ECG (HR, 2.31 [95%CI, 0.88–6.08]; p = 0.091) (Fig. 3D).

## 4 Discussion

This post hoc analysis of a large randomized trial has demonstrated in a prospective, real-world, patient population with suspected cardiac chest pain, that (1) CMR has better overall diagnostic performance than exercise ECG, (2) SPECT provides greater specificity and positive predictive value than exercise ECG, and (3) CMR alone as the first-line test was more sensitive and prognostically significant than exercise ECG, SPECT, or a sequential combination of both of these tests.

The clinical utility of the exercise ECG compared to functional imaging is important to determine given its widespread use, despite extensive evidence from randomized trials, registries, meta-analyses, and systematic reviews 8, 9, 17, 18 showing limited diagnostic yield to detect obstructive CAD. Albeit recognizing its reduced accuracy compared to stress imaging, current guidelines still recommend the use of exercise ECG in low-risk patients, as well as in patients with intermediate or high risk who demonstrate high exercise capacity (≥5 metabolic equivalents), to rule out myocardial ischemia, assess the patient’s functional capacity, and for risk stratification purposes [6]. This study adds to the existing body of evidence for exercise ECG in a prospectively recruited population in which 73% of the patients were at intermediate risk and 80% had an interpretable ECG and were considered able to exercise. The diagnostic performance of the exercise ECG in this population was similar to previously published data. Importantly, contrary to historical belief, no sex differences existed in this population, with comparable diagnostic accuracy of exercise ECG in males and females 1, 18. These results help close a knowledge gap regarding the diagnostic accuracy of exercise ECG in women [19].

Direct diagnostic and prognostic comparisons of exercise ECG with other cardiac imaging tests are lacking [10]. In the present head-to-head comparison, pharmacological stress imaging had better diagnostic performance than exercise ECG. CMR on its own outperformed exercise ECG on all diagnostic accuracy measures, while SPECT was either better (specificity and PPV) or comparable (sensitivity and NPV) to exercise ECG. The poor prognostic performance of the exercise ECG observed in this trial reflects findings from other trials, including from the ISCHEMIA, where the extent of ST segment depression was not found to be predictive of the trial’s primary endpoint, nor of CV death or myocardial infarction [20].

Furthermore, in this trial, only 13% of those able to exercise had an unequivocally normal exercise ECG and 44% had an inconclusive test result. Using SPECT or CMR to adjudicate the inconclusive exercise ECG results is reflective of typical cardiology practice and recommended in current US guidelines [6]. Comparison of a strategy of exercise ECG+CMR with exercise ECG+SPECT in this analysis revealed that exercise ECG+CMR had superior sensitivity and NPV, likely reflecting the superior diagnostic accuracy of CMR alone as previously found in the main CE-MARC trial [12]. The strategy of exercise ECG+SPECT also demonstrated improved sensitivity and NPV compared to SPECT alone, but with the penalty of significantly worse specificity and PPV. However, when the two combination strategies were compared on their prognostic yield in both patients with an interpretable or inconclusive exercise ECG, the strategy of exercise ECG+CMR outperformed that of exercise ECG+SPECT, as sequential SPECT had limited additional discriminative ability.

In this analysis, a strategy of CMR alone was the optimal diagnostic pathway and resulted in the greatest potential reduction in unnecessary coronary angiography even when baseline CAD risk was considered (Supplementary Material and Fig. S3). Neither SPECT on its own or in combination with exercise ECG outperformed CMR first-line for the diagnosis of obstructive CAD. However, a strategy of exercise ECG followed by CMR in patients with inconclusive results could be considered potentially equivalent, in terms of diagnostic accuracy and prognostic ability, to CMR alone, and therefore might have clinical utility in health care systems with reduced access to CMR. In particular, a strategy of exercise ECG+CMR compared to CMR on its own produced a small increase in sensitivity, at a penalty of a large reduction in specificity and positive PPV. The same was found with regard to risk stratification, where a strategy of CMR alone was more prognostically significant than a strategy of exercise ECG+CMR.

Importantly, our trial design did not allow us to assess the potential impact of microvascular dysfunction on trial results. Recent work showed 100% specificity for the exercise ECG to detect coronary microvascular dysfunction [21] among patients with angina and non-obstructive coronary arteries (ANOCA) on coronary computed tomography (CT). However, this was accompanied by poor rule-in ability (sensitivity of 41%) and poor diagnostic accuracy (∼ 55%) in a selected cohort of patients where microvascular dysfunction was highly prevalent at 77% (pooled prevalence of coronary microvascular dysfunction in ANOCA being 41% [22]). More broadly, it should be noted that the exercise ECG offers significant clinical information in terms of symptom assessment, often allowing the referring physician to pinpoint the cardiac origin of chest pain or equivalents.

The cost-effectiveness of the exercise ECG, SPECT, and CMR alone and in combination in the CE-MARC trial has previously been reported [23]. A diagnostic strategy in which CMR follows a positive or inconclusive exercise ECG, followed by coronary angiography if CMR is positive or inconclusive and a strategy in which CMR is used as a first-line test were both considered cost-effective from a UK health care perspective. Although the data suggest that a combination of exercise ECG followed by SPECT is useful in increasing testing sensitivity in the current analysis, it was not cost-effective in the health economic analysis. In CE-MARC, CMR as a first-line test was both the most accurate and cost-effective diagnostic strategy. Similarly, a post hoc analysis of the Stress CMR Perfusion Imaging in the United States study showed that, among patients with intermediate risk, a CMR-based management strategy was clinically effective in the US health care system, and superior to invasive angiography, SPECT, and coronary CT angiography from a cost-effectiveness standpoint [24].

Since the CE-MARC trial was published, several methodological advancements have been reached [25] in both SPECT and CMR. The availability of high-resolution perfusion images, as well as the possibility to quantitate myocardial blood flow at rest and stress [26], along with the implementation of artificial intelligence algorithms in the interpretation of imaging studies [27], were not available at the time the CE-MARC trial was conducted. Despite their potential, widespread adoption of these newer methods remains limited. For this reason, we believe our findings continue to apply to a large majority of health care providers worldwide. Technical advances likely improve imaging modalities’ diagnostic and prognostic performance in patients with chest pain. Future studies should elucidate their incremental value over widely available, standard methods.

## 5 Limitations

This was a post hoc subanalysis of a prospective randomized trial in patients with suspected stable cardiac chest pain. This might reduce the generalizability of our results, although patients in the present study were not statistically or clinically different at baseline from those enrolled in the main trial. This study did not address the possible influence of coronary microvascular dysfunction and ANOCA on the diagnostic and prognostic evaluation of the performed tests, and thus its results may not be generalizable to other patient populations. Finally, the study inherits the main trial limitations, including low ethnic diversity and reliance on an anatomical reference for obstructive CAD, which did not include the assessment of fractional flow reserve.

## 6 Conclusions

In patients with suspected stable cardiac chest pain, exercise ECG was less sensitive than stress CMR and less specific than stress CMR and SPECT alone. CMR had higher diagnostic and prognostic yield than exercise ECG, SPECT, or a combination of the two. A strategy of exercise ECG followed by CMR in patients with an inconclusive exercise ECG result provided comparable accuracy and could have clinical utility in health care settings where CMR is less accessible. Given its proven diagnostic, prognostic, and cost-effectiveness, wider adoption of CMR as the first-line functional test in patients with chest pain and suspected angina appears warranted.

## Funding

10.13039/501100000274British Heart Foundation (RG/05/004).

## Author contributions

**Chiara Bucciarelli-Ducci:** Writing – review & editing, Investigation, Conceptualization. **Sven Plein:** Writing – review & editing. **Julia M. Brown:** Writing – review & editing. **Giandomenico Bisaccia:** Writing – review & editing, Writing – original draft, Methodology, Investigation, Formal analysis, Data curation. **John P. Greenwood:** Writing – review & editing, Methodology, Investigation, Funding acquisition, Formal analysis, Conceptualization. **Neil Maredia:** Writing – review & editing. **Catherine J. Dickinson:** Writing – review & editing. **Peter P. Swoboda:** Writing – review & editing, Supervision. **John F. Younger:** Writing – review & editing.

## Declaration of competing interests

C.B.D. is the CEO (part-time) of the Society for Cardiovascular Magnetic Resonance (SCMR) and received lecture fees from GE HealthCare, Circle Cardiovascular Imaging, Bayer, and Siemens Healthineers. J.P.G. received lecture fees from Bayer and Edwards Lifesciences. Other authors declare no relationships relevant to the contents of this paper.

## Data Availability

Data utilized in the present study will be shared upon reasonable request to the corresponding author.

## Footnotes

Appendix A Supplementary data associated with this article can be found in the online version at doi:10.1016/j.jocmr.2025.101960.
