# Supplementary material for: Diagnostic and prognostic comparison of stress electrocardiogram, cardiovascular magnetic resonance, and single photon emission computed tomography, alone and sequentially, in stable chest pain
Source: J Cardiovasc Magn Reson. 2025 Sep 15;27(2):101960. doi: 10.1016/j.jocmr.2025.101960 (PMC12745148; doi:10.1016/j.jocmr.2025.101960)
Supplement: Supplementary file 1 — Supplementary material [file mmc1.docx]

**Supplementary Material**

[Supplementary Results 2](#_Toc209510759)

[Diagnostic accuracy of exercise ECG by sex 2](#_Toc209510760)

[Diagnostic accuracy of exercise ECG by pre-test risk category 2](#_Toc209510761)

[Diagnostic accuracy of the Exercise ECG for detection of left main stem and triple vessel disease 2](#_Toc209510762)

[Diagnostic accuracy of the Exercise ECG Duke Treadmill Score 3](#_Toc209510763)

[Diagnostic accuracy of CMR and SPECT in the exercise ECG subgroup compared to whole cohort 3](#_Toc209510764)

[Diagnostic accuracy of exercise ECG versus CMR and SPECT with scored results 3](#_Toc209510765)

[Prognostic value of exercise ECG by sex 3](#_Toc209510766)

[Prognostic value of exercise ECG by pre-test risk category 4](#_Toc209510767)

[Prognostic value of the Exercise ECG Duke Treadmill Score 4](#_Toc209510768)

[Supplementary Tables 5](#_Toc209510769)

[Table S1. Extent of significant coronary heart disease in male and female patients having exercise ECG and coronary angiography (n=580). 5](#_Toc209510770)

[Table S2. Exercise ECG findings. 6](#_Toc209510771)

[Table S3. Diagnostic accuracy of exercise ECG, CMR and SPECT in the exercise ECG population (compared to CMR and SPECT results from the main trial). 7](#_Toc209510772)

[Table S4. Diagnostic accuracy of exercise ECG, CMR and SPECT (with inconclusive exercise ECGs as negative). 8](#_Toc209510773)

[Table S5. Diagnostic accuracy of exercise ECG combined with CMR or with SPECT (accepting positive and negative exercise ECG results and using CMR or SPECT to adjudicate in those with inconclusive findings). 9](#_Toc209510774)

[Table S6. Exercise Electrocardiography Parameters by MACE Status. 10](#_Toc209510775)

[Supplementary Figures 13](#_Toc209510776)

[Figure S1. Diagnostic accuracy of exercise ECG with inconclusive tests considered as negative and for the subgroup with inconclusive tests removed. 13](#_Toc209510777)

[Figure S2. Diagnostic accuracy of exercise ECG, CMR and SPECT with scored results. 14](#_Toc209510778)

[Figure S3. Clinical utility of exercise ECG, CMR and SPECT when baseline CAD risk is considered. 15](#_Toc209510779)

[CONSORT Checklist 16](#_Toc209510780)

## Supplementary Results

### Diagnostic accuracy of exercise ECG by sex

Sensitivity and specificity of exercise ECG were similar in men and women (**Table S3)**. However, there was markedly lower PPV (more false positives) in women compared to men and correspondingly higher NPV (fewer false negatives) reflecting the much lower prevalence of significant coronary disease in women (20%) compared to men (49%).

### Diagnostic accuracy of exercise ECG by pre-test risk category

We separately assessed the diagnostic accuracy of exercise ECG in patients at low and intermediate-high pre-test likelihood of CAD according to current published estimates (Juarez-Orozco et al. Eur Heart Journal 2019). Among patients with angiographic and exercise ECG results available, 452 (78%) were considered at intermediate-high risk, while 128 (22%) were at low risk. Among patients at low CAD risk, the sensitivity, specificity, PPV and NPV of exercise ECG were 75%, 75%, 23.7% and 96.7%, respectively. Among patients with a PTP > 15%, the sensitivity, specificity, PPV and NPV of exercise ECG were 67.9%, 71.3%, 67.6%, and 71.6%, respectively. The AUC for detection of significant CAD did not differ significantly between patients at low and intermediate-high risk (70.2 vs 68.9%, p>0.05).

### Diagnostic accuracy of the Exercise ECG for detection of left main stem and triple vessel disease

We assessed if exercise ECG results could identify patients with left main stem (LMS) or three-vessel disease. Among patients with the exercise ECG available, N = 17 patients had LMS disease. Among these, 14 (82%) had a positive exercise ECG (p < 0.001), corresponding to a sensitivity of 60%, specificity of 79%, PPV of 98%, and NPV of 12% (AUC 70%). N = 33 patients had triple vessel disease. Of these, 26 (79%) had a positive exercise ECG (p < 0.001), corresponding to a sensitivity of 82%, specificity of 59%, PPV of 99%, and NPV of 6.3% (AUC 69%).

### Diagnostic accuracy of the Exercise ECG Duke Treadmill Score

Duke Treadmill Score (DTS) was available in 570 (98%) out of 580 patients having the exercise ECG. With a reference of invasive angiography, DTS had an AUC of 76.81%. At a threshold of -4.5, DTS had a sensitivity of 61.5% and a specificity of 79.7%, with PPV of 66.0% and NPV of 76.4%.

### Diagnostic accuracy of CMR and SPECT in the exercise ECG subgroup compared to whole cohort

The overall sensitivities and specificities for CMR and SPECT in the whole population have been previously published^12^ and are available in **Table S3 C&E**. Of the 580 patients having exercise ECG and angiography, 539 also had CMR and 520 had SPECT. As expected, the findings in these two subgroups (Table S3 D&F) were similar to the overall main study results (**Table S3 C&E**).

### Diagnostic accuracy of exercise ECG versus CMR and SPECT with scored results

For scored results ROC analysis, SPECT and CMR studies were labelled as: 1) normal, 2) probably normal, 3) compatible with IHD, 4) compatible with mild inducible ischaemia, 5) compatible with moderate/severe inducible ischaemia, or 6) inconclusive. Exercise ECG studies were labelled as either positive, borderline positive, negative, or inconclusive. At ROC curve comparison, CMR was found to allow better CAD discrimination than exercise ECG (p < 0.0001) and SPECT (p < 0.0001). Contrary to main ROC analysis, in this analysis of scored test results SPECT was found to be significantly more accurate than exercise ECG (p = 0.0061) (**Figure S2**).

### Prognostic value of exercise ECG by sex

At a median follow-up of 6.9 years (IQR 0.8-7.3), 64 male (17.2%) and 27 female (12.9%) patients experienced a MACE. Exercise ECG was not prognostic in males (HR 0.89, 95%CI 0.55-1.46, log-rank p = 0.7), nor in females (HR 1.75, 95%CI 0.82-3.75, log-rank p = 0.15).

### Prognostic value of exercise ECG by pre-test risk category

6 patients at low risk of CAD (1%) and 61 at intermediate-high risk (10.5%) experienced a MACE. While Exercise ECG was prognostic for low-risk patients (log-rank p = 0.025), it failed to risk stratify patients at a CAD PTP > 15% (log-rank p = 0.62).

### Prognostic value of the Exercise ECG Duke Treadmill Score

The prognostic value of the DTS was assessed for time to first MACE. DTS was not prognostic (HR 0.97, 95%CI 0.93-1.00, log-rank p = 0.06). DTS-based risk classification was also assessed. Patients were considered at 1) low risk of CAD if they achieved a DTS greater than 4, 2) moderate risk if they had a DTS between -10 and 4 included, and 3) high risk for a DTS lower than -10. This classification was also not prognostic (log-rank p = 0.31).

## Supplementary Tables

### Table S1. Extent of significant coronary heart disease in male and female patients having exercise ECG and coronary angiography (n=580).

| Variable |  |  | Male Patients | Female Patients |
| --- | --- | --- | --- | --- |
| Patients with any significant coronary artery stenosis: | | | 184 (49%) | 40 (20%) |
| Triple Vessel Disease |  |  | 34 (9%) | 4 (2%) |
| Double Vessel Disease |  |  | 60 (16%) | 10 (5%) |
| Single Vessel Disease |  |  | 90 (24%) | 26 (13%) |
| LMS Disease |  |  | 16 (4%) | 4 (1%) |
| LAD Disease |  |  | 131 (35%) | 21 (10%) |
| LCX Disease |  |  | 88 (23%) | 17 (8%) |
| RCA Disease |  |  | 73 (19%) | 13 (6%) |

### Table S2. Exercise ECG findings.

|  | **Mean (SD or %)** |
| --- | --- |
| Patients (n) | 601 |
| Resting HR (bpm) | 76.5 (14.2) |
| Resting systolic BP (mmHg) | 135.4 (19.4) |
| Resting diastolic BP (mmHg) | 77.5 (10.8) |
| Maximal HR (bpm) | 143.2 (22.5) |
| Maximal systolic BP (mmHg) | 174.9 (27.9) |
| Maximal diastolic BP (mmHg) | 79.3 (12.8) |
| Change in HR (bpm) | 66.7 (20.7) |
| Change in systolic BP (mmHg) | 39.5 (22.9) |
| Change in diastolic BP (mmHg) | 1.8 (10.2) |
| Achieved 85% of predicted HR* | 389 (64.9%) |
| Minimal recovery HR (bpm) | 116.1 (23.0) |
| HR recovery (bpm) | 27.0 (11.8) |
| METs | 9.1 (3.3) |
| Any symptoms during exercise | 373 (62.3%) |
| Duke treadmill score | -3.0 (6.0) |

* by age and sex

### Table S3. Diagnostic accuracy of exercise ECG, CMR and SPECT in the exercise ECG population (compared to CMR and SPECT results from the main trial).

| **TEST** | **Number of patients** | **Sensitivity, % (95% CI)** | **Specificity, % (95% CI)** | **Positive Predictive Value, % (95% CI)** | **Negative Predictive Value, % (95% CI)** |
| --- | --- | --- | --- | --- | --- |
| A. Exercise ECG (inconclusive tests considered negative) | 580 | 68.3 | 72.5 | 61.0 | 78.4 |
|  |  | (61.9, 74.0) | (67.6, 76.9) | (54.8, 66.8) | (73.7, 82.5) |
| Males | 375 | 67.4 | 68.6 | 67.4 | 68.6 |
|  |  | (60.3, 73.7) | (61.7, 74.7) | (60.3, 73.7) | (61.7, 74.7) |
| Females | 205 | 72.5 | 77.0 | 43.3 | 92.0 |
|  |  | (57.2, 83.9) | (70.0, 82.7) | (32.1, 55.2) | (86.3, 95.5) |
|  | M&F | P=0.5291 | P=0.0774 | * | * |
| B. Exercise ECG (inconclusive tests excluded) | 324 | 96.8 | 41.0 | 61.0 | 93.2 |
|  |  | (92.8, 98.6) | (33.8, 48.6) | (54.8, 66.8) | (84.9, 97.0) |
|  |  |  |  |  |  |
| C. CMR (main trial) | 676 | 86.5 | 83.4 | 77.2 | 90.5 |
|  |  | (81.8, 90.1) | (79.5, 86.7) | (72.1, 81.6) | (87.1, 93.0) |
| D. CMR (in exercise ECG group) | 539 | 87.2 | 86.9 | 81.1 | 91.3 |
|  |  | (82.0, 91.1) | (82.8, 90.1) | (75.5, 85.6) | (87.7, 94.0) |
| E. SPECT (main trial) | 676 | 66.5 | 82.6 | 71.4 | 79.1 |
|  |  | (60.4-72.1) | (78.5-86.1) | (65.3-76.9) | (74.8-82.8) |
| F. SPECT (in exercise ECG group) | 520 | 65.7 | 84.0 | 72.1 | 79.5 |
|  |  | (58.9, 71.9) | (79.6, 87.6) | (65.2, 78.1) | (74.9, 83.5) |

*the negative and positive predictive values are not compared statistically because of the large difference in prevalence of disease between males and females.

### Table S4. Diagnostic accuracy of exercise ECG, CMR and SPECT (with inconclusive exercise ECGs as negative).

| **TEST** | **AUC (95%CI)** |
| --- | --- |
| CMR | 84.52 (81.32-87.71) |
| Exercise ECG | 70.39 (66.55-74.22) |
| SPECT | 73.88 (70.06-77.69) |
| **ROC curves comparison** (Delong method) | |
| CMR vs exercise ECG: p <0.0001 CMR vs SPECT: p <0.0001 SPECT vs exercise ECG: p = 0.241 | |

### Table S5. Diagnostic accuracy of exercise ECG combined with CMR or with SPECT (accepting positive and negative exercise ECG results and using CMR or SPECT to adjudicate in those with inconclusive findings).

| **TEST** | **Number of patients** | **Sensitivity, % (95% CI)** | **Specificity, % (95% CI)** | **Positive Predictive Value, % (95% CI)** | **Negative Predictive Value, % (95% CI)** |
| --- | --- | --- | --- | --- | --- |
| A. Exercise ECG (+ & -) combined with CMR | 503 | 91.9 | 66.0 | 63.5 | 92.7 |
|  |  | (87.2, 94.9) | (60.5, 71.1) | (57.8, 68.9) | (88.4, 95.4) |
| B. exercise ECG (+ & -) combined with SPECT | 503 | 86.3 | 64.1 | 60.7 | 87.9 |
|  |  | (80.8, 90.4) | (58.5, 69.2) | (54.9, 66.3) | (83.0, 91.5) |
|  | A&B | P=0.0266 | P=0.4177 | P=0.0607 | P=0.0114 |
| C. CMR | 503 | 86.8 | 86.6 | 80.7 | 91.1 |
|  |  | (81.4-90.8) | (82.3-90.0) | (74.8-85.4) | (87.2-93.8) |
|  | exercise ECG+CMR vs. CMR alone (A&C) | P=0.0414 | P<0.0001 | P<0.0001 | P=0.3209 |
| D. SPECT | 503 | 65.5 | 83.7 | 72.1 | 79.0 |
|  |  | (58.6-71.8) | (79.1-87.4) | (65.1-78.1) | (74.3-83.1) |
|  | exercise ECG+SPECT vs. SPECT alone (B&D) | P<0.0001 | P<0.0001 | P=0.0085 | P<0.0001 |

A strategy of sequential CMR after exercise ECG had significantly superior sensitivity and NPV compared to sequential SPECT after exercise ECG (P=0.0266 and P=0.0114 respectively), but the specificities and PPV’s were similar (**A&B**). A strategy of sequential SPECT after exercise ECG improved the sensitivity compared to SPECT alone (86.3%&65.5%) but at a cost of specificity, which fell from 83.7% to 64.1% (**D&B**). Correspondingly the NPV was improved from 79.0% to 87.9% but with a fall in the PPV from 72.1% to 60.7% (**D&B**). For sequential use of CMR after exercise ECG, compared to CMR alone, there was a small increase in sensitivity from 86.8% to 91.9% with a reduction in specificity from 86.6% to 66.0% (**C&A**). The combination of exercise ECG and CMR compared to CMR alone resulted in comparable NPV but with a significant reduction in the PPV from 80.7% to 63.5% (**C&A**).

### Table S6. Exercise Electrocardiography Parameters by MACE Status.

| **Exercise ECG Parameters** | **Overall** N = 582*^1^* | **Major Adverse Cardiac Events (MACE)** | | **P-value***^2^* |
| --- | --- | --- | --- | --- |
|  |  | **No MACE** N = 491*^1^* | **MACE** N = 91*^1^* |  |
| Normal Resting ECG | 22 (71.0%) | 21 (84.0%) | 1 (16.7%) | **0.004** |
| Left Bundle Branch Block | 0 (0.0%) | 0 (0.0%) | 0 (0.0%) |  |
| Right Bundle Branch Block | 1 (7.1%) | 0 (0.0%) | 1 (25.0%) | 0.29 |
| Left Ventricular Hypertrophy | 4 (26.7%) | 0 (0.0%) | 4 (80.0%) | **0.004** |
| Non-specific ST Changes | 4 (30.8%) | 3 (30.0%) | 1 (33.3%) | >0.99 |
| T Wave Changes | 4 (30.8%) | 3 (30.0%) | 1 (33.3%) | >0.99 |
| Anterior Q Wave | 0 (0.0%) | 0 (0.0%) | 0 (0.0%) |  |
| Inferior Q Waves | 1 (7.7%) | 1 (10.0%) | 0 (0.0%) | >0.99 |
| Lateral Q Waves | 0 (0.0%) | 0 (0.0%) | 0 (0.0%) |  |
| ETT Test Completed | 581 (99.8%) | 490 (99.8%) | 91 (100.0%) | >0.99 |
| Exercise Protocol |  |  |  | **0.001** |
| Bruce Protocol | 568 (97.8%) | 484 (98.8%) | 84 (92.3%) |  |
| Modified Bruce Protocol | 13 (2.2%) | 6 (1.2%) | 7 (7.7%) |  |
| Resting Heart Rate (bpm) | 76 (65.0, 86) | 76 (65.0, 86) | 75 (66.0, 86) | 0.97 |
| Resting Systolic BP (mmHg) | 136 (120.0, 150) | 134 (120.0, 148) | 140 (129.0, 152) | **0.015** |
| Resting Diastolic BP (mmHg) | 80 (70.0, 84) | 80 (70.0, 84) | 80 (70.0, 87) | 0.15 |
| Peak Heart Rate (bpm) | 144 (127.5, 160) | 146 (128.0, 161) | 139 (124.0, 154) | **0.017** |
| Peak Systolic BP (mmHg) | 174 (158.0, 194) | 172 (160.0, 190) | 179 (150.5, 205) | 0.19 |
| Peak Diastolic BP (mmHg) | 80 (70.0, 87) | 80 (70.0, 86) | 80 (70.0, 90) | 0.30 |
| Achieved ≥85% Predicted HR | 377 (65.0%) | 326 (66.7%) | 51 (56.0%) | 0.056 |
| Exercise Duration (minutes) | 6 (4.0, 8) | 6 (4.0, 8) | 5 (3.0, 7) | **0.005** |
| Exercise Duration (seconds) | 29 (14.0, 43) | 28 (13.0, 43) | 31 (18.5, 46) | 0.10 |
| Total Exercise Duration (minutes) | 6 (4.4, 9) | 6 (4.6, 9) | 5 (3.8, 8) | **0.008** |
| Peak METs Achieved | 10 (7.0, 10) | 10 (7.0, 10) | 7 (7.0, 10) | **0.001** |
| Clinical Symptoms Reproduced | 361 (62.2%) | 300 (61.3%) | 61 (67.0%) | 0.35 |
| Exercise-Induced Angina |  |  |  | 0.88 |
| Absent | 239 (41.2%) | 202 (41.3%) | 37 (40.7%) |  |
| Limiting | 122 (21.0%) | 101 (20.7%) | 21 (23.1%) |  |
| Non-limiting | 219 (37.8%) | 186 (38.0%) | 33 (36.3%) |  |
| Primary Reason for Test Termination |  |  |  | **0.021** |
| Arrhythmia | 6 (1.0%) | 3 (0.6%) | 3 (3.3%) |  |
| Chest pain | 102 (17.6%) | 84 (17.2%) | 18 (19.8%) |  |
| Fatigue | 136 (23.4%) | 120 (24.5%) | 16 (17.6%) |  |
| Hypertension | 12 (2.1%) | 9 (1.8%) | 3 (3.3%) |  |
| Hypotension | 8 (1.4%) | 8 (1.6%) | 0 (0.0%) |  |
| Leg Pain | 21 (3.6%) | 13 (2.7%) | 8 (8.8%) |  |
| Other | 67 (11.6%) | 60 (12.3%) | 7 (7.7%) |  |
| SOB | 151 (26.0%) | 125 (25.6%) | 26 (28.6%) |  |
| ST changes | 63 (10.9%) | 53 (10.8%) | 10 (11.0%) |  |
| Target heart rate achieved | 14 (2.4%) | 14 (2.9%) | 0 (0.0%) |  |
| Maximum ST Depression (mm) | 1 (0.6, 2) | 1 (0.6, 2) | 1 (0.8, 2) | 0.70 |
| Time to Max ST Deviation (minutes) | 6 (4.0, 8) | 6 (4.0, 8) | 5 (3.0, 7) | **0.012** |
| Time to Max ST Deviation (seconds) | 30 (14.0, 44) | 30 (14.0, 44) | 30 (12.3, 42) | 0.78 |
| 1-Minute Recovery HR (bpm) | 117 (99.0, 135) | 119 (99.8, 135) | 111 (95.3, 127) | **0.050** |
| Heart Rate Recovery (bpm) | 26 (20.0, 34) | 26 (20.0, 34) | 24 (17.3, 33) | 0.20 |
| Duke Treadmill Score | -2 (-6.8, 1) | -2 (-6.8, 1) | -3 (-7.6, 1) | 0.17 |
| Duke Score | -2 (-6.8, 1) | -2 (-6.8, 1) | -3 (-7.6, 1) | 0.17 |

## Supplementary Figures

### Figure S1. Diagnostic accuracy of exercise ECG with inconclusive tests considered as negative and for the subgroup with inconclusive tests removed.


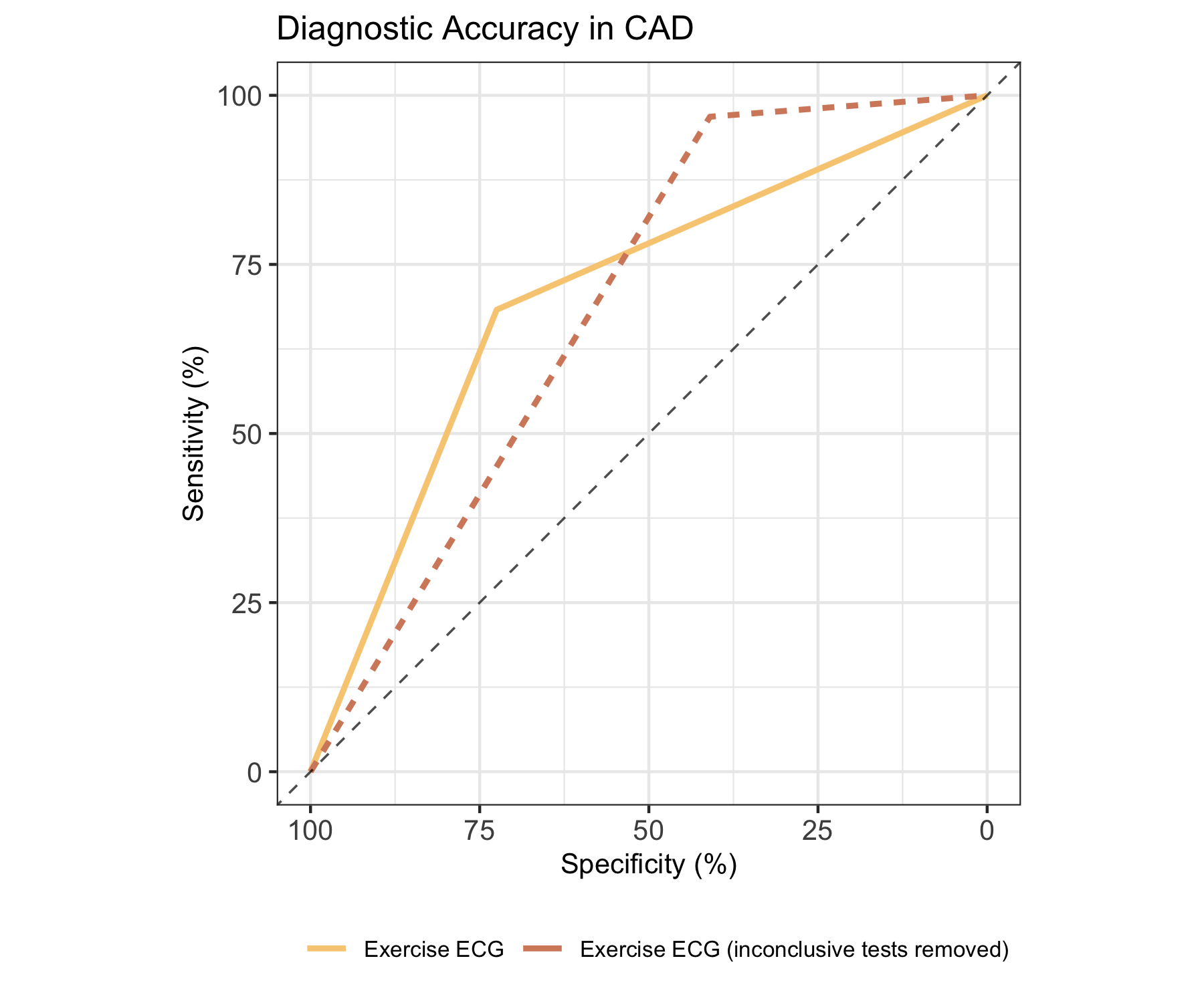


### Figure S2. Diagnostic accuracy of exercise ECG, CMR and SPECT with scored results.


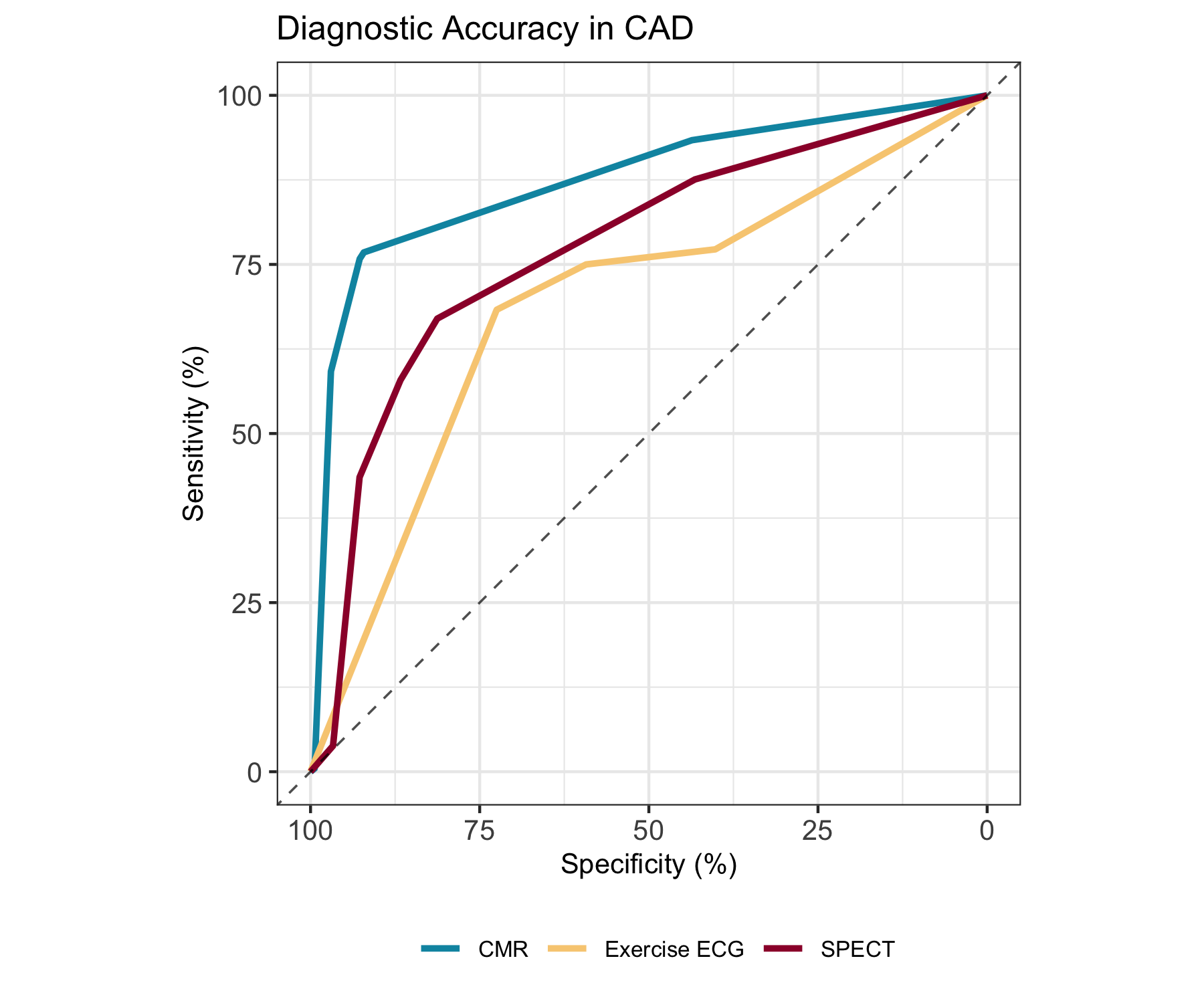


### Figure S3. Clinical utility of exercise ECG, CMR and SPECT when baseline CAD risk is considered.


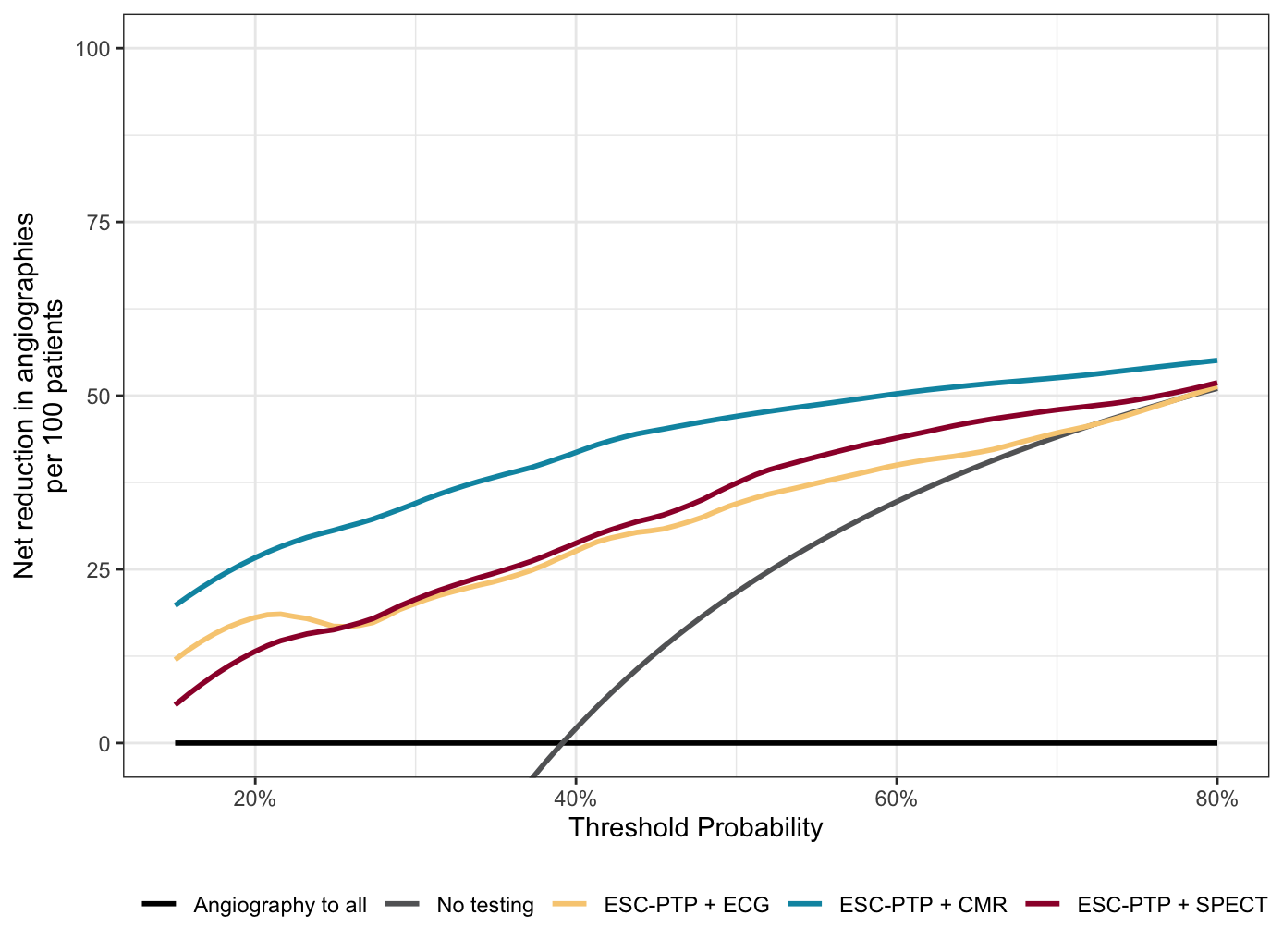


The net reduction in invasive coronary angiographies is displayed according to each modality’s diagnostic performance. Patients’ baseline risk of CAD is based on estimates from the ESC-PTP model (<https://doi.org/10.1093/ehjci/jez054>) for patients with chronic coronary syndromes. CMR is incremental to the ESC-PTP score along the full preference spectrum and may lead to 20% to 50% reduction in unnecessary invasive coronary angiographies, depending on the threshold used for referral. When combined with baseline risk stratification by the ESC-PTP score, CMR offered the most consistent clinical utility for avoiding unnecessary invasive angiographies, with SPECT and exercise ECG providing comparatively smaller albeit measurable benefits.

## CONSORT Checklist

|  |  | **Reporting Item** | **Page Number** |
| --- | --- | --- | --- |
| **Title and Abstract** |  |  |  |
| Title | [#1a](https://www.goodreports.org/reporting-checklists/consort/info/#1a) | Identification as a randomized trial in the title. | 1 |
| Abstract | [#1b](https://www.goodreports.org/reporting-checklists/consort/info/#1b) | Structured summary of trial design, methods, results, and conclusions | 2 |
| **Introduction** |  |  |  |
| Background and objectives | [#2a](https://www.goodreports.org/reporting-checklists/consort/info/#2a) | Scientific background and explanation of rationale | 4 |
| Background and objectives | [#2b](https://www.goodreports.org/reporting-checklists/consort/info/#2b) | Specific objectives or hypothesis | 4-5 |
| **Methods** |  |  |  |
| Trial design | [#3a](https://www.goodreports.org/reporting-checklists/consort/info/#3a) | Description of trial design (such as parallel, factorial) including allocation ratio. | 5 |
| Trial design | [#3b](https://www.goodreports.org/reporting-checklists/consort/info/#3b) | Important changes to methods after trial commencement (such as eligibility criteria), with reasons | n/a |
| Participants | [#4a](https://www.goodreports.org/reporting-checklists/consort/info/#4a) | Eligibility criteria for participants | 6 |
| Participants | [#4b](https://www.goodreports.org/reporting-checklists/consort/info/#4b) | Settings and locations where the data were collected | 6 |
| Interventions | [#5](https://www.goodreports.org/reporting-checklists/consort/info/#5) | The experimental and control interventions for each group with sufficient details to allow replication, including how and when they were actually administered | 6-8 |
| Outcomes | [#6a](https://www.goodreports.org/reporting-checklists/consort/info/#6a) | Completely defined prespecified primary and secondary outcome measures, including how and when they were assessed | 8-9 |
| Sample size | [#7a](https://www.goodreports.org/reporting-checklists/consort/info/#7a) | How sample size was determined. | CE-MARC trial protocol |
| Sample size | [#7b](https://www.goodreports.org/reporting-checklists/consort/info/#7b) | When applicable, explanation of any interim analyses and stopping guidelines | n/a |
| Randomization - Sequence generation | [#8a](https://www.goodreports.org/reporting-checklists/consort/info/#8a) | Method used to generate the random allocation sequence. |  |
| 7 |  |  |  |
| Randomization - Sequence generation | [#8b](https://www.goodreports.org/reporting-checklists/consort/info/#8b) | Type of randomization; details of any restriction (such as blocking and block size) |  |
| CE-MARC trial protocol |  |  |  |
| Randomization - Allocation concealment mechanism | [#9](https://www.goodreports.org/reporting-checklists/consort/info/#9) | Mechanism used to implement the random allocation sequence (such as sequentially numbered containers), describing any steps taken to conceal the sequence until interventions were assigned | n/a |
| Randomization - Implementation | [#10](https://www.goodreports.org/reporting-checklists/consort/info/#10) | Who generated the allocation sequence, who enrolled participants, and who assigned participants to interventions | n/a |
| Blinding | [#11a](https://www.goodreports.org/reporting-checklists/consort/info/#11a) | If done, who was blinded after assignment to interventions (for example, participants, care providers, those assessing outcomes) and how. | 8 |
| Blinding | [#11b](https://www.goodreports.org/reporting-checklists/consort/info/#11b) | If relevant, description of the similarity of interventions | n/a |
| Statistical methods | [#12a](https://www.goodreports.org/reporting-checklists/consort/info/#12a) | Statistical methods used to compare groups for primary and secondary outcomes | 8-9 |
| Statistical methods | [#12b](https://www.goodreports.org/reporting-checklists/consort/info/#12b) | Methods for additional analyses, such as subgroup analyses and adjusted analyses | 8-9 |
| Outcomes | [#6b](https://www.goodreports.org/reporting-checklists/consort/info/#6b) | Any changes to trial outcomes after the trial commenced, with reasons | n/a |
| **Results** |  |  |  |
| Participant flow diagram (strongly recommended) | [#13a](https://www.goodreports.org/reporting-checklists/consort/info/#13a) | For each group, the numbers of participants who were randomly assigned, received intended treatment, and were analysed for the primary outcome | 19 |
| Participant flow | [#13b](https://www.goodreports.org/reporting-checklists/consort/info/#13b) | For each group, losses and exclusions after randomization, together with reason | n/a |
| Recruitment | [#14a](https://www.goodreports.org/reporting-checklists/consort/info/#14a) | Dates defining the periods of recruitment and follow-up | 6,  Published protocol,  Main trial |
| Recruitment | [#14b](https://www.goodreports.org/reporting-checklists/consort/info/#14b) | Why the trial ended or was stopped | n/a |
| Baseline data | [#15](https://www.goodreports.org/reporting-checklists/consort/info/#15) | A table showing baseline demographic and clinical characteristics for each group | 21-22 |
| Numbers analysed | [#16](https://www.goodreports.org/reporting-checklists/consort/info/#16) | For each group, number of participants (denominator) included in each analysis and whether the analysis was by original assigned groups | 11 |
| Outcomes and estimation | [#17a](https://www.goodreports.org/reporting-checklists/consort/info/#17a) | For each primary and secondary outcome, results for each group, and the estimated effect size and its precision (such as 95% confidence interval) | 9-14 |
| Outcomes and estimation | [#17b](https://www.goodreports.org/reporting-checklists/consort/info/#17b) | For binary outcomes, presentation of both absolute and relative effect sizes is recommended | 9-14 |
| Ancillary analyses | [#18](https://www.goodreports.org/reporting-checklists/consort/info/#18) | Results of any other analyses performed, including subgroup analyses and adjusted analyses, distinguishing pre-specified from exploratory | 12-14; Supplementary Material |
| Harms | [#19](https://www.goodreports.org/reporting-checklists/consort/info/#19) | All important harms or unintended effects in each group (For specific guidance see CONSORT for harms) | n/a |
| **Discussion** |  |  |  |
| Limitations | [#20](https://www.goodreports.org/reporting-checklists/consort/info/#20) | Trial limitations, addressing sources of potential bias, imprecision, and, if relevant, multiplicity of analyses | n/a |
| Interpretation | [#22](https://www.goodreports.org/reporting-checklists/consort/info/#22) | Interpretation consistent with results, balancing benefits and harms, and considering other relevant evidence | 14-17 |
| Registration | [#23](https://www.goodreports.org/reporting-checklists/consort/info/#23) | Registration number and name of trial registry | 3 |
| Generalisability | [#21](https://www.goodreports.org/reporting-checklists/consort/info/#21) | Generalisability (external validity, applicability) of the trial findings | 14-17 |
| **Other information** |  |  |  |
| Interpretation | [#22](https://www.goodreports.org/reporting-checklists/consort/info/#22) | Interpretation consistent with results, balancing benefits and harms, and considering other relevant evidence | 15-18 |
| Registration | [#23](https://www.goodreports.org/reporting-checklists/consort/info/#23) | Registration number and name of trial registry | 2 |
| Protocol | [#24](https://www.goodreports.org/reporting-checklists/consort/info/#24) | Where the full trial protocol can be accessed, if available | 5 |
| Funding | [#25](https://www.goodreports.org/reporting-checklists/consort/info/#25) | Sources of funding and other support (such as supply of drugs), role of funders | 18 |
